# Vorinostat, temozolomide or bevacizumab with irradiation and maintenance BEV/TMZ in pediatric high-grade glioma: A Children’s Oncology Group Study

**DOI:** 10.1093/noajnl/vdae035

**Published:** 2024-03-14

**Authors:** Rishi R Lulla, Allen Buxton, Mark D Krailo, Margot A Lazow, Daniel R Boue, James L Leach, Tong Lin, James I Geller, Shiva Senthil Kumar, Marina N Nikiforova, Uma Chandran, Sachin S Jogal, Marvin D Nelson, Arzu Onar-Thomas, Daphne A Haas-Kogan, Kenneth J Cohen, Mark W Kieran, Amar Gajjar, Rachid Drissi, Ian F Pollack, Maryam Fouladi

**Affiliations:** Department of Pediatrics, Hasbro Children’s Hospital, The Warren Alpert Medical School of Brown University, Providence, Rhode Island, USA; Department of Biostatistics, Children’s Oncology Group, Monrovia, California, USA; Department of Biostatistics, Children’s Oncology Group, Monrovia, California, USA; Pediatric Neuro‑Oncology Program, Nationwide Children’s Hospital, The Ohio State University College of Medicine, Columbus, Ohio, USA; Department of Pathology and Laboratory Medicine, Nationwide Children’s Hospital, The Ohio State University College of Medicine, Columbus, Ohio, USA; Department of Radiology and Medical Imaging, Cincinnati Children’s Hospital Medical Center, Department of Radiology, University of Cincinnati College of Medicine, Cincinnati, Ohio, USA; Department of Biostatistics, St. Jude Children’s Research Hospital, Memphis, Tennessee, USA; Division of Oncology, Cincinnati Children’s Hospital Medical Center, University of Cincinnati, Cincinnati, Ohio, USA; Center for Childhood Cancer Research, Nationwide Children’s Hospital, Columbus, Ohio, USA; Division of Molecular & Genomic Pathology, University of Pittsburgh Medical Center, Pittsburgh, Pennsylvania, USA; Department of Biomedical Informatics, University of Pittsburgh, Pittsburgh, Pennsylvania, USA; Department of Pediatrics, Medical College of Wisconsin, Milwaukee, Wisconsin, USA; Department of Radiology, Children’s Hospital Los Angeles, Keck University of Southern California School of Medicine, Los Angeles, California, USA; Department of Biostatistics, St. Jude Children’s Research Hospital, Memphis, Tennessee, USA; Department of Radiation Oncology, Brigham and Women’s Hospital, Boston Children’s Hospital, Dana-Farber Cancer Institute, Harvard Medical School, Boston, Massachusetts, USA; Division of Pediatric Oncology, Department of Pediatrics, The Johns Hopkins University School of Medicine, Baltimore, Maryland, USA; Department of Pediatric Oncology, Dana-Farber/Boston Children’s Cancer and Blood Disorders Center, Harvard Medical School, Boston, Massachusetts, USA; Department of Pediatric Medicine, St Jude Children’s Research Hospital, Memphis, Tennessee, USA; Center for Childhood Cancer Research, Nationwide Children’s Hospital, Columbus, OH, The Ohio State University College of Medicine, Columbus, Ohio, USA; Department of Neurosurgery, UPMC Children’s Hospital of Pittsburgh, University of Pittsburgh, Pittsburgh, Pennsylvania, USA; Pediatric Neuro‑Oncology Program, Nationwide Children’s Hospital, The Ohio State University College of Medicine, Columbus, Ohio, USA

**Keywords:** bevacizumab, high-grade glioma, pediatric, radiation, telomerase

## Abstract

**Background:**

Outcomes for children with high-grade gliomas (HGG) remain poor. This multicenter phase II trial evaluated whether concurrent use of vorinostat or bevacizumab with focal radiotherapy (RT) improved 1-year event-free survival (EFS) compared to temozolomide in children with newly diagnosed HGG who received maintenance temozolomide and bevacizumab.

**Methods:**

Patients ≥ 3 and < 22 years with localized, non-brainstem HGG were randomized to receive RT (dose 54–59.4Gy) with vorinostat, temozolomide, or bevacizumab followed by 12 cycles of bevacizumab and temozolomide maintenance therapy.

**Results:**

Among 90 patients randomized, the 1-year EFS for concurrent bevacizumab, vorinostat, or temozolomide with RT was 43.8% (±8.8%), 41.4% (±9.2%), and 59.3% (±9.5%), respectively, with no significant difference among treatment arms. Three- and five-year EFS for the entire cohort was 14.8% and 13.4%, respectively, with no significant EFS difference among the chemoradiotherapy arms. *IDH* mutations were associated with more favorable EFS (*P* = .03), whereas H3.3 K27M mutations (*P* = .0045) and alterations in *PIK3CA* or *PTEN* (*P* = .025) were associated with worse outcomes. Patients with telomerase- and alternative lengthening of telomeres (ALT)-negative tumors (*n* = 4) had an EFS of 100%, significantly greater than those with ALT or telomerase, or both (*P* = .002). While there was no difference in outcomes based on *TERT* expression, high *TERC* expression was associated with inferior survival independent of the telomere maintenance mechanism (*P* = .0012).

**Conclusions:**

Chemoradiotherapy with vorinostat or bevacizumab is not superior to temozolomide in children with newly diagnosed HGG. Patients with telomerase- and ALT-negative tumors had higher EFS suggesting that, if reproduced, mechanism of telomere maintenance should be considered in molecular-risk stratification in future studies.

**Key Points**

Chemoradiotherapy with bevacizumab or vorinostat does not improve outcomes in pediatric high-grade gliomas (HGG).
Mechanism of telomere maintenance may impact outcomes in pediatric HGG.

**Importance of the Study**

In this prospective, randomized, multi-centered phase II clinical trial, we validated the biological and clinical diversity of pediatric high-grade glioma (HGG) demonstrated in prior clinical trials and large retrospective series, and provided long-term survival data. Addition of bevacizumab or vorinostat as chemoradiotherapy and use of temozolomide and bevacizumab as maintenance therapy did not improve event-free survival (EFS) compared to chemoradiotherapy with temozolomide alone. In our cohorts, patients with H3.3 K27M mutations (*P* = .0045) and alterations in *PIK3CA* or *PTEN* (*P* = .025) were associated with worse outcomes. This study also prospectively evaluated the role, prognostic significance, and co-occurring genetic alterations of telomere maintenance mechanisms in pediatric HGG. A subgroup of patients with telomerase- and alternative lengthening of telomeres (ALT)-negative tumors had significantly improved EFS, suggesting that mechanisms of telomere maintenance should be considered as part of molecular-risk stratification in future studies.

Despite multimodal therapy with surgery, radiation therapy (RT), and chemotherapy, outcomes for children with high-grade glioma (HGG) remain dismal.^1–3^ After maximal surgical resection, conventional treatment for newly diagnosed patients consists of either RT alone or with concurrent temozolomide and may include adjuvant temozolomide and/or nitrosourea based on existing adult and pediatric clinical trial data.^4,5^

There is a critical need for novel, effective, and well-tolerated therapeutic strategies for pediatric HGG, including those that potentiate the RT effect and augment the temozolomide backbone. Dysregulated histone acetylation as well as vascular endothelial growth factor overexpression have been implicated in tumorigenesis of pediatric HGGs.^6,7^ Furthermore, emerging preclinical and clinical data at the time of the present trial’s development suggested potential antitumor activity and RT synergy of histone deacetylase (HDAC) and vascular endothelial growth factor inhibition in this disease, providing rationale for investigating vorinostat and bevacizumab, respectively.^6,7^ Additionally, the combination of bevacizumab and temozolomide had yielded promising early results in recurrent adult HGG.^8^ The Children’s Oncology Group (COG) ACNS0822 phase II study sought to evaluate the efficacy of chemoradiotherapy with vorinostat or bevacizumab compared to concurrent temozolomide, all with a maintenance backbone of temozolomide and bevacizumab.

This trial was designed and opened to enrollment in the era before contemporary molecular characterization. Subsequent discoveries have provided valuable insight into the underlying biology of pediatric HGGs.^9–12^ Genome-wide sequencing analyses have revealed recurring mutations in a subset of midline HGGs in genes encoding histone H3.3 (*H3-3A*, previously called *H3F3A*) or H3.1 (*H3C2,* previously called *HIST1H3B*), resulting in key substitution of lysine to methionine at position 27 (K27M) as well as glycine to arginine or valine at position 34 in non-midline tumors (G34R/V), with subsequent aberrant transcription and associated prognostic significance.^13–15^ Additional somatic alterations of receptor tyrosine kinase, cell-cycle regulation, DNA repair, and/or PI3K/AKT/mTOR signaling pathways have been identified within molecularly distinct subgroups of pediatric HGG,^9–11^ with therapeutic and prognostic implications. Although stratification by these molecular biomarkers was not possible at the time ACNS0822 was designed, targeted sequencing and relevant immunohistochemistry were performed retrospectively in a large subset of patients, offering an opportunity to confirm the biologic heterogeneity and predictors of outcome within pediatric HGG in a multi-institutional, prospective setting. Furthermore, given increased evidence implicating telomere dysregulation in gliomagenesis,^16–18^ this trial sought to prospectively investigate the role, prognostic relevance, and co-occurring genetic alterations of telomerase maintenance mechanisms in pediatric HGG.

Here, we report the results of the randomized, multi-centered phase II study ACNS0822 (**ClinicalTrials.gov ID** NCT01236560) to determine whether concurrent vorinostat (dosing determined through an initial feasibility cohort) or bevacizumab administered with focal RT would improve 1-year event-free survival (EFS) compared to temozolomide in children newly diagnosed with HGG who all received maintenance therapy with bevacizumab and temozolomide. Secondary objectives include evaluating EFS and overall survival (OS) for the entire cohort who received the bevacizumab and temozolomide maintenance backbone; focused molecular profiling and assessment of telomerase maintenance mechanisms; further characterizing toxicity; and describing patterns of progression.

## Methods

### Study Design

The COG ACNS0822 study was a prospective phase II/III, open-label, randomized, multicenter trial of vorinostat, temozolomide, or bevacizumab with local RT followed by maintenance with bevacizumab and temozolomide for children with newly diagnosed HGG. The combination of vorinostat with local RT had not previously been tested in children; therefore, an initial 6-patient feasibility study was performed to determine the safe and tolerable dose of vorinostat in this setting.

This study was designed and executed according to the principles outlined in the World Medical Association Declaration of Helsinki. After central and local institutional review board approval, eligible patients were consented in person, and study treatment began within 31 days after definitive surgery. Intracranial tumors received RT at a dose of 54.0 Gy to the preoperative tumor volume plus a 2 cm margin in 1.8 Gy fractions if a gross-total resection (GTR) was performed. For incomplete resections, residual disease was boosted to a total dose of 59.4 Gy. Primary spinal cord tumors received a dose of 50.4–54.0 Gy in 1.8 Gy fractions regardless of resection extent.

The primary objective of the feasibility study was to identify the recommended phase II dose (RP2D) of vorinostat when administered in combination with focal RT in patients with newly diagnosed HGG. Vorinostat was administered at the single agent pediatric RP2D of 230 mg/m^2^ 5 days per week for 6 weeks, with a planned dose de-escalation to 180 mg/m^2^/day, 5 days per week for 6 weeks if the single agent RP2D proved intolerable. The randomized phase II portion of the study included chemoradiotherapy with vorinostat (at the RP2D determined in the feasibility study), temozolomide (90 mg/m^2^/day daily for 42 days), or bevacizumab (10 mg/kg/dose on days 22 and 36). Four weeks after the completion of RT, all patients proceeded to maintenance chemotherapy with bevacizumab (10 mg/kg/dose every 2 weeks) and temozolomide (200 mg/m^2^/day, days 1–5 of each 4-week cycle) for up to 12 cycles. The phase II portion employed a randomized “pick-the-winner” approach to determine if either of 2 experimental treatment arms (bevacizumab or vorinostat during chemoradiotherapy) would yield a higher 1-year EFS than the standard treatment arm (temozolomide during chemoradiotherapy). In that scenario, the study would be expanded to a 2-arm phase III trial between the “winner” from the phase II portion and the standard arm.

### Eligibility

Patients aged ≥ 3 and < 22 years with newly diagnosed, localized, intracranial or spinal cord WHO 2007 grade III and IV HGG including anaplastic astrocytoma, glioblastoma, or gliosarcoma were enrolled. Note that histopathologic diagnosis for eligibility relied on local pathology reports; a retrospective central pathology review was subsequently completed (Table 1). Preoperative and post-operative disease site MRIs with gadolinium were required for study entry. Other eligibility criteria included Karnofsky or Lansky performance score ≥ 50%, no prior cancer therapy, and adequate organ function. Key exclusion criteria included diagnosis of oligodendroglioma or oligoastrocytoma; primary brainstem tumors; metastatic HGG as defined by imaging evidence of neuro-axis dissemination or positive CSF cytology; known bleeding diathesis, coagulopathy, or thrombophilic condition; history of arterial or deep venous thromboembolic events; evidence of significant post-operative intracranial hemorrhage; uncontrolled hypertension; major surgical procedure within 7 days prior to planned start of therapy; uncontrolled seizures; use of enzyme-inducing anti-convulsants; or history of a prior malignancy.

**Table 1.** Baseline Patient Characteristics of Patients Enrolled on the Phase II Portion of ACNS0822

	Vorinostat (*N* = 31)	Bevacizumab (*N* = 32)	Temozolomide (*N* = 27)	ALL patients (*N* = 96)
*Sex*				
Female	15 (48.4%)	12 (37.5%)	14 (51.9%)	44 (45.8%)
Male	16 (51.6%)	20 (62.5%)	13 (48.1%)	52 (54.2%)
*Age at enrollment*				
Median (Range)	12.4 (4.1–20.7)	11.8 (3.4–20.0)	12.5 (4.3–22.0)	12.2 (3.4–22.0)
*Age category*				
<5 years	1 (3.2%)	4 (12.5%)	1 (3.7%)	6 (6.3%)
≥10, <15 years	12 (38.7%)	8 (25.0%)	10 (37.0%)	33 (34.4%)
≥5, <10 years	5 (16.1%)	9 (28.1%)	8 (29.6%)	23 (24.0%)
≥15 years	13 (41.9%)	11 (34.4%)	8 (29.6%)	34 (35.4%)
*Central pathology review* ^*^				
Astrocytoma, NOS	0	0	1 (3.7%)	1 (1.0%)
Astrocytoma, anaplastic	9 (29.0%)	13 (40.6%)	8 (29.6%)	32 (33.3%)
Ependymoma, anaplastic	0	1 (3.1%)	0	1 (1.0%)
Giant cell glioblastoma	1 (3.2%)	0	0	1 (1.0%)
Glioblastoma, NOS	18 (58.1%)	15 (46.9%)	14 (51.9%)	51 (53.1%)
Glioma, malignant	3 (9.7%)	1 (3.1%)	3 (11.1%)	7 (7.3%)
Gliosarcoma	0	2 (6.3%)	0	2 (2.1%)
Missing	0	0	1 (3.7%)	1 (1.0%)
*Simplified disease classification*				
Anaplastic astrocytoma (AA)*	12 (38.7%)	12 (37.5%)	11 (40.7%)	37 (38.5%)
Glioblastoma multiforme (GBM)	19 (61.3%)	20 (62.5%)	16 (59.3%)	59 (61.5%)
*Extent of tumor resection*				
Biopsy only	6 (19.4%)	8 (25.0%)	8 (29.6%)	23 (24.0%)
Gross total resection	8 (25.8%)	10 (31.3%)	10 (37.0%)	28 (29.2%)
Near total resection	7 (22.6%)	5 (15.6%)	4 (14.8%)	17 (17.7%)
Partial resection	4 (12.9%)	3 (9.4%)	2 (7.4%)	11 (11.5%)
Subtotal resection	6 (19.4%)	6 (18.8%)	3 (11.1%)	17 (17.7%)
*Location*				
Cerebral hemisphere	10 (32.3%)	12 (37.5%)	11 (40.7%)	34 (35.4%)
Basal ganglia-diencephalon	6 (19.4%)	8 (25.0%)	5 (18.5%)	20 (20.8%)
Brainstem	1 (3.2%)	0	1 (3.7%)	2 (2.1%)
Cerebellar	12 (38.7%)	12 (37.5%)	10 (37.0%)	38 (39.6%)
Spinal cord	2 (6.5%)	0	0	2 (2.1%)

^*^Histopathologic diagnoses determined from local pathology reports were used for eligibility. Retrospective central pathology review was subsequently performed, with results presented in the table. The patient with ependymoma was excluded from subsequent analyses.

### Study Assessment

Tumor evaluations were performed at baseline (post-operative, if surgery was performed), 4 weeks after the completion of chemoradiotherapy, every 8 weeks during maintenance therapy, and every 3 months thereafter until disease progression and/or recurrence. COG guidelines for tumor measurement based on cross-sectional imaging were applied. Criteria for response included: complete response: disappearance of all target lesions; partial response: ≥50% decrease in the sum of the products of the 2 perpendicular diameters; progressive disease: ≥25% increase in the product of perpendicular diameters of any lesion. Recommendations for recognition of pseudo-progression in the first 3 months after RT were made to avoid removing patients from protocol therapy prematurely.

### Toxicities and Dose Modifications

Adverse events were graded according to the National Cancer Institute Common Toxicity Criteria, version 4.0. The COG Data Safety Monitoring Committee provided oversight for the study. Dose-modifying toxicities were defined as any of the following events at least possibly attributable to therapy: Grade 3 thrombocytopenia that requires transfusion > 2 times during RT; grade 4 neutropenia or thrombocytopenia; grade 4 non-hematological toxicity; or grade 3 non-hematological toxicity with the specific exclusion of grade 3 nausea and vomiting < 5 days duration, grade 3 increased alanine aminotransferase (ALT) that returns to eligibility levels within 7 days of drug disruption and does not recur upon rechallenge, grade 3 fever or infection < 5 days, and grade 3 electrolyte abnormalities that are responsive to oral supplementation.

### Correlative Studies

Consenting patients provided optional tumor specimens for gene expression and targeted mutation analysis, *MGMT* methylation assessment, measurement of telomerase activity, *hTERT* expression and TERC RNA levels, telomere length, and ALT evaluation.

#### Gene expression and activation.—

Immunohistochemical analysis was used to assess the expression and activation of proteins that were associated with outcomes in prior CCG (Children’s Cancer Group) and COG studies. Tissue sections were deparaffinized and rehydrated; after blocking endogenous peroxidase activity and antigen retrieval,^19^ sections were incubated with primary antibodies against pAkt (Ser^473^; Cell Signaling, 1:50); p53 (DO-7, Dako Corporation, Carpinteria, CA, 1:300); pMAPK (Thr^202^/Tyr^204^; Cell Signaling, 1:200); MIB1 (Immunotech, Westbrook, ME; USA 1:100); and MGMT (mT3.1, Chemicon International, Temecula, CA, 1:400). Slides were rinsed with PBS (phosphate-buffered saline) and incubated with biotinylated anti-mouse or anti-rabbit IgG (Vector, 1:200) followed by streptavidin-horseradish peroxidase conjugate (Vector Elite). Antibody binding was visualized using 3,3^’^-diaminobenzidine (DAB). Slides were counterstained, dehydrated through graded concentrations of ethanol, cleared in xylene, mounted, and examined using a light microscope. Labeling was graded as positive or negative as previously described.^19,20^ For pAKT, pMAPK, and p53, tumors with more than 10% positive cells, either focally or diffusely within the lesion, were categorized as exhibiting overexpression in relation to normal brain. MIB1 labeling was directly quantitated into high, medium, or low ranges as previously reported.^21^ MGMT labeling was semi-quantitatively assessed as described in prior CCG/COG studies.^1,3,5^

#### Nucleic acid isolation.—

For FFPE tissues, tumor-rich areas (≥50% neoplastic cells) were micro-dissected from unstained histologic sections under guidance of a hematoxylin & eosin stained (H&E) slide using an Olympus SZ61 microscope (Olympus, Hamburg, Germany). Total nucleic acids (TNA) were isolated with the DNeasy Blood and Tissue kit on the QIAcube (Qiagen, Valencia, CA). For frozen tumors, TNA isolation was performed using the MagNA Pure LC Total Nucleic Acid Isolation Kit (Roche, Indianapolis, IN). Extracted DNA and RNA were quantitated on the Qubit 2.0 Fluorometer (Invitrogen).

#### Loss of heterozygosity analysis.—

Loss of heterozygosity (LOH) analysis was performed as previously described.^19^ First, DNA was amplified using primers flanking microsatellite regions adjacent to target genes of interest (eg, *CDKN2A*, *PTEN*, and *TP53*, among others). PCR amplification products were analyzed by capillary gel electrophoresis on an ABI 3730 platform (Applied Biosystems). The relative fluorescence values (peak heights) were measured using GeneScan 3.7 software (Applied Biosystems) and a 1.5-fold or greater difference in peak height ratios between alleles in tumor and normal specimens was indicative of LOH.

#### Targeted next-generation sequencing.—

GlioSeq amplification-based targeted next-generation sequencing (NGS) analysis was performed using 5–10 ng of DNA and RNA as previously described.^22^ Custom GlioSeq NGS libraries were prepared to detect SNVs and small insertions/deletions in 30 key brain tumor genes, for copy number changes in 24 genes, and > 20 types of gene fusions.^22^ The sequencing was performed using an Ion Proton instrument (Thermo Fisher Scientific) according to the manufacturer’s instructions. DNA sequencing data was analyzed using Torrent Suite software (version 4.4.3) and Variant Caller (Thermo Fisher Scientific), and further analyzed using an internally created software suite. The analytical sensitivity of GlioSeq is 3-5% of mutant alleles for detection of SNVs and indels, 30% of tumor nuclei for detection of CNAs, and 1% of cells with fused transcript for gene fusions. The minimum required sequencing depth is 300×. Confirmation of mutations was performed by Sanger sequencing. Copy number changes for amplification targets (eg, *EGFR*, *PDGFRA*, and *MET*) and fusions (eg, *BRAF*) were confirmed by fluorescence in situ hybridization or, for *EGFRvIII*, by reverse transcription (RT)-PCR.^23,24^

#### Analysis of MGMT promoter methylation.—

MGMT methylation was assessed by real-time (MethyLite) PCR and by MSP and agarose gel electrophoresis as previously reported.^25–27^ Samples showing strong amplification by MethyLite PCR and a visible band on MSP were considered methylated.

#### Assessment of telomerase activity, ALT status, hTERT and TERC expression and TERT promoter mutations.—

Telomerase activity, ALT status by telomere restriction fragment analysis and mRNA levels of *hTERT* and *TERC* expression were analyzed as previously described.^18^ To confirm ALT status, C-circle assay was performed based on previously described methods.^28^ Briefly, following φ29 amplification, DNA was transferred to a charged nylon membrane and detection was carried out using the TeloTAGGG kit (Roche Diagnostics, Indianapolis, IN) following manufacturer’s instructions for telomere detection. Samples showing positive signal were considered ALT positive while those with no signal were ALT negative. For *hTERT* promoter mutations (C228T and C250T), DNA was extracted as previously described.^18^ A Region of the *hTERT* promoter harboring the recurrent mutations C228T and C250T was amplified using forward (5ʹ-AGCACCTCGCGGTAGTGG-3ʹ) and reverse (5ʹ-GTCCTGCCCCTTCACCTT-3ʹ) primers followed by Sanger sequencing of the amplified product.

### Study Objectives and Endpoints

For the feasibility portion of the study, the primary endpoint was the identification of a tolerable dose of vorinostat in combination with RT in pediatric patients with newly diagnosed HGG. One-year EFS (time to the first occurrence of disease progression, relapse, second malignant neoplasm, or death from any cause) was the primary efficacy endpoint of the phase II randomized portion of the study. Secondary objectives included estimation of EFS and OS, evaluation of toxicities on each of the treatment arms, assessment of telomerase activity, *hTERT* expression TERC RNA levels, ALT activity, and telomere length as well as targeted gene mutation and expression analysis in newly diagnosed pediatric HGG.

### Statistical Design and Analysis Plan

The feasibility portion of the study planned to enroll 6 patients at 230 mg/m^2^/dose of vorinostat with daily RT. If fewer than 2 patients experienced DLTs, this dose would be declared as feasible and would be carried forward to the randomized component. Otherwise, a dose de-escalation to 180 mg/m^2^/dose was planned.

For the randomized phase II component, a “pick-the-winner design” was implemented based on simulations where the arm with the superior 1-year EFS among the 2 experimental arms would be carried forward if its 1-year EFS was also higher than the 1-year EFS observed on the control arm (chemoradiotherapy with temozolomide). Assuming a 1-year EFS of 45% for the control arm (consistent with the ACNS0126 and ACNS0423 outcomes available at the time) and 106 patients evenly randomized across the 3 arms, this design was estimated to have reasonable power (>70%) to identify the “promising” experimental arm if the difference in 1-year EFS between the 2 experimental arms was at least 10%.

If a “winner” was selected, the phase III portion of the trial would be randomized between the “winner” and the control arm, and the primary comparison would be based on a log-rank test comparing the EFS distributions. Again, based on simulations, the sample size was estimated as 100 subjects per arm including those enrolled on the phase II portion. The planned log-rank comparison would use 5% type 1 error and would be conducted 1-year post accrual completion.

The study also incorporated a formal monitoring rule for toxic death and grade 3/4 hemorrhage (Supplementary Data). Outcome distributions were estimated by using the Kaplan-Meier method. Two-sided Log-rank tests were used for outcome comparisons. Fisher’s exact test was applied to compare distributions of categorical variables among patient groups. Statistical analyses were done using R-3.4.0 and no multiplicity adjustment was used.

## Results

### Patients

Between November 2010 and April 2014, 101 patients were enrolled in ACNS0822. Five patients were deemed ineligible due to timing of enrollment (*n* = 1), missing pre-study labs (*n* = 3), and lack of tissue for central review (*n* = 1). Six patients were enrolled in the feasibility study (vorinostat + RT) and the remaining 90 patients were randomized for the phase II portion of the study (vorinostat + RT [*n* = 31]; bevacizumab + RT [*n* = 32]; temozolomide + RT [*n* = 27]). Baseline characteristics among treatment groups in the phase II study were similar and are shown in Table 1. All survival outcome analyses were limited to the patients enrolled on the randomized phase II portion of the study.

### Feasibility Study (Vorinostat + RT)

The 6 patients enrolled in the feasibility portion of the protocol received RT with vorinostat at 230 mg/m^2^/day for 5 days per week for a total of 6 weeks during RT. All patients were evaluable for toxicity, and none experienced a DLT, such that the phase II dose for vorinostat with irradiation was determined to be 230 mg/m^2^/dose.

### Primary Efficacy Endpoint

**Figure 1.**
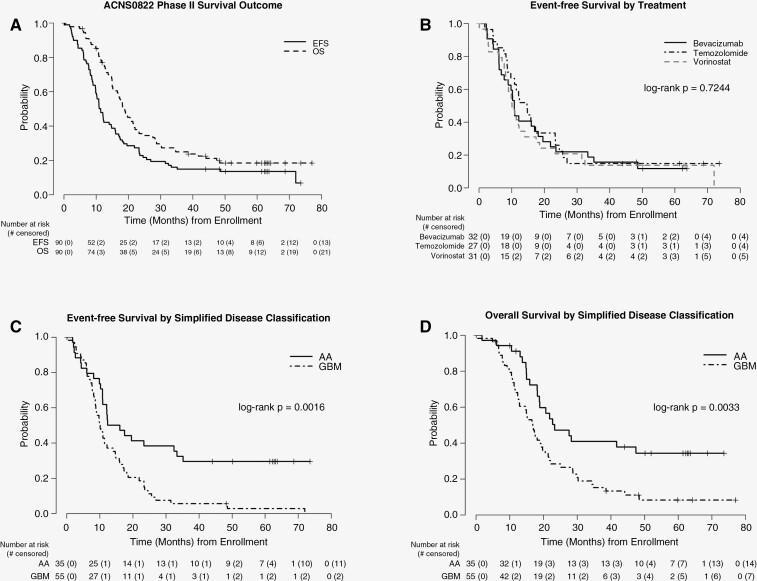
Overall and event-free survival for all patients in the phase II portion of ACNS0822 (A). Event-free survival by chemoradiotherapy treatment arm (B). Event-free survival (C) and overall survival (D) by simplified disease classification. The number of patients at risk over time is shown below the curves.

As shown in Figure 1A and Table 2, the 1-year EFS and OS for all patients enrolled in the phase II portion of ACNS0822 is 47.7% (±5.3%) and 76.9% (±4.5%), respectively. The 1-year EFS rates for chemoradiotherapy with bevacizumab, vorinostat, or temozolomide are 43.8% (± 8.8%), 41.4% (± 9.2%) and 59.3% (± 9.5%), respectively. There was no significant difference in EFS distributions across the 3 treatment arms (*P* = .7244, Figure 1B). Similarly, no significant difference in OS was observed (*P* = .6348). Thus, the study closed to accrual following a Data Safety Monitoring Committee recommendation due to lack of efficacy slightly short of the planned accrual.

**Table 2.** Survival Data for ACNS0822 and Other Recently Published HGG Clinical Trials

	1-year EFS	1-year OS	2-year EFS	2-year OS	3-year EFS	3-year OS	5-year EFS	5-year OS
ACNS0126^1^	37%	69%	17%	29%	11%	20%	8%	12%
ACNS0423^5^	49%	72%	30%	46%	22%	28%	14%	22%
ACNS0822 ALL	47.7%	76.9%	22.7%	35.5%	14.8%	24.9%	13.4%	18.4%
ACNS0822 TMZ + RT	59.3%	85.2%	25.9%	37.0%	14.8%	25.9%	14.8%	16.7%
ANCS0822 VOR + RT	41.4%	82.2%	20.7%	28.6%	13.8%	21.5%	13.8%	14.3%
ACNS0822 BEV + RT	43.8%	65.0%	21.9%	41.1%	15.6%	27.4%	11.7%	23.9%
HERBY RT + BEV + TMZ^29^	38%	68%						
HERBY RT + TMZ^29^	48%	75%						

As shown in Table 2, long-term survival data is available for patients from the phase II portion.

For the entire cohort, 2-year, 3-year, and 5-year EFS estimates are 22.7% (± 4.5%), 14.8% (± 3.8%), and 13.4% (± 3.7%), respectively. Two-year, 3-year, and 5-year OS estimates are 35.5% (± 5.2%), 24.9% (± 4.7%), and 18.4% (± 4.3%), respectively.

When outcomes were evaluated by histology regardless of treatment arm, patients with glioblastoma had significantly poorer EFS (*P* = .0016) and OS (*P* = .0033) compared to patients with anaplastic astrocytoma, astrocytoma NOS, giant cell glioblastoma and gliosarcoma (Figure 1C and D). For the entire cohort, there was no difference in outcomes based on extent of surgery (*P* = .3906), though patients with anaplastic astrocytoma who achieved a GTR had improved EFS (*P* = .0155) compared to patients with glioblastoma who achieved a GTR.

### Patterns of Progression

We characterized the pattern of progression (local, distant, or combined) for all patients (*n* = 80) who progressed during the study. While most patients had local progression, 29 patients (36%) across all arms demonstrated a distant or combined pattern of failure. There was no difference in the observed pattern of failure among the 3 treatment arms.

### Adverse Events

Treatment in this study was well tolerated. There were no dose-limiting toxicities in the feasibility study of vorinostat with local irradiation. In the phase II portion of the study, patients who received temozolomide during chemoradiotherapy had a higher incidence of grade 3 or 4 toxicities including hematologic toxicities and fever when compared to those treated with vorinostat or bevacizumab. Patients who received vorinostat during chemoradiotherapy had a higher incidence of nausea and vomiting. A single patient in the phase II study had a grade 4 thromboembolic event during chemoradiotherapy with vorinostat. During maintenance therapy, the most common grade 3 or 4 toxicities for all patients were hematologic toxicities with no difference observed among patients treated on the 3 chemoradiotherapy arms. No patients had grade 3 or 4 thromboembolic events during maintenance, and a single patient had a grade 3 intracranial hemorrhage during maintenance with bevacizumab.

### Biologic Features

For patients enrolled in the phase II portion of the study who consented to optional molecular testing and had evaluable specimens, we explored the association of each biological variable with survival outcomes as shown in Table 3. *H3-3A* mutations were associated with inferior 1-year EFS and OS and *IDH* mutations were associated with improved outcomes. There was no significant difference in 1-year EFS and OS for the immunohistochemical assessment of MIB-1, p53, MGMT, pAKT, or pMAPK, or analysis of *MGMT* promoter methylation status. Copy number analysis revealed LOH at chromosome 10q and chromosome 17p, the loci for *PTEN* and *TP53*, respectively, was associated with poorer 1-year EFS and OS.

**Table 3.** Association of Biological Variables with Survival for Patients Enrolled on ACNS0822

	Log-rank test *P*-value	
Biological variable	1-year EFS	1-year OS
MIB-1 by IHC (*n* = 52; 17 or less vs. 18–36 vs. more than 36)	.1179	.2955
p53 by IHC (*n* = 69; negative vs. positive)	.6751	.7607
MGMT by IHC (*n* = 69; negative vs. positive)	.8645	.7541
pat by IHC (*n* = 66; negative vs. positive)	.4736	.1927
pMAPK (*n* = 67; negative vs. positive)	.6485	.7046
Chr1p LOH (*n* = 57; yes vs. no)	.6978	.5651
Chr9p LOH (*n* = 53; yes vs. no)	.1115	.2279
Chr10q LOH (*n* = 51; yes vs. no)	.0321	.008
Chr17p LOH (*n* = 46; yes vs. no)	.0063	.0006
Chr19q LOH (*n* = 55; yes vs. no)	.4184	.2458
*MGMT* (*n* = 58; methylated vs. unmethylated)	.8700	.7609
*H3-3A* K27M (*n* = 57; mutated vs. wild type)	.0588	.0706
*H3-3A* G34 (*n* = 57; mutated vs. wild type)	.0476	.0999
*H3-3A* K27M or G34 combined (*n* = 57; K27M or G34 vs. wild type)	.0019	.0055
*TP53* mutation (*n* = 57; mutated vs. wild type)	.4223	.1536
*IDH* mutation (*n* = 58; mutated vs. wild type)	.0239	.0663
*ATRX* mutation (*n* = 57; mutated vs. wild type)	.7579	.781
*PIK3CA*/*PTEN* mutation (*n* = 57; mutated vs. wild type)	.0464	.3709
*EGFR* mutation/amplification (*n* = 57; mutated vs. wild type)	.3205	.4005
*MET* mutation/amplification (*n* = 57; mutated vs. wild type)	.7472	.6494
*CDKN2A*/*RB* mutation (*n* = 57; mutated vs. wild type)	.0812	.1017

**Figure 2.**
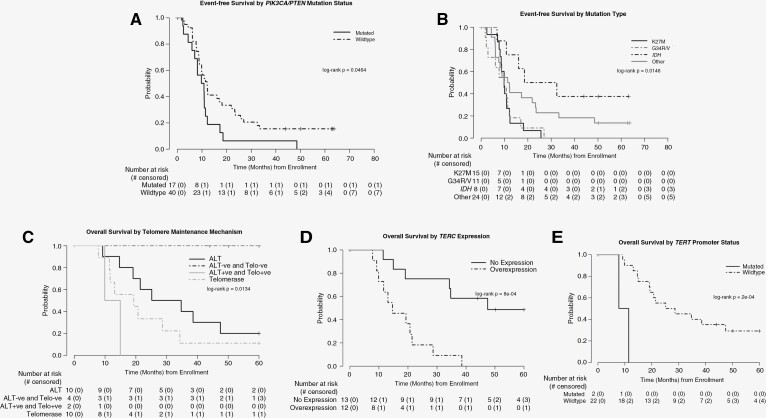
Biological variables impact survival on ACNS0822. Event-free survival by *PIK3CA/PTEN* mutation status (A) and *H3-3A* and *IDH* mutation status (B). Overall survival by telomere maintenance mechanism (C), *TERC* expression (D), and *TERT* Promoter status (E). The number of patients at risk over time is shown below the curves.

Targeted NGS profiling in evaluable specimens (*n* = 57) demonstrated that *H3-3A* mutations were mutually exclusive with *IDH* mutations in this cohort. Patients with tumors that harbored *H3-3A* K27M mutations (*n* = 15) had inferior EFS (*P* = .05) compared to patients with H3K27-wild-type tumors (*n* = 42). As a group, patients with *H3-3A* K27M or G34R/V mutant tumors (*n* = 26) had significantly poorer outcomes (EFS and OS) when compared to patients with *H3-3A*-wild-type tumors (*P* < .006). Patients with *PIK3CA* or *PTEN* mutations (*n* = 17) also had significantly worse EFS compared to those with wild-type tumors (*n* = 40; *P* = .0464). In contrast, patients with *IDH*-mutant tumors (*n* = 8) had improved EFS (*P* = .0239) compared to patients with *IDH*-wild-type tumors. Survival data by selected mutation types is shown in Figure 2.

### Telomere Maintenance in Pediatric HGG

A total of 31 patients submitted specimens to evaluate the prognostic significance of telomere maintenance in pediatric HGG. Tumors were grouped by mechanism of telomere maintenance: telomerase activity only, ALT only, both telomerase and ALT, or unidentified telomere maintenance mechanism (ALT- and telomerase-negative). We then performed survival analysis on 30 patients with complete telomere maintenance data enrolled in the phase II portion of the study. As shown in Figure 2C, patients with tumors that were both telomerase and ALT negative (*n* = 4) had an EFS and OS of 100%, significantly different from those with ALT only, telomerase only, or both (*P* = .0134).

While there was no difference in outcomes based on *TERT* expression in our cohort, high levels of *TERC* expression were associated with lower EFS and OS (Figure 2D). Two patients with *TERT* promoter mutations C228T had a significant increase in *hTERT* expression and were noted to have inferior outcomes compared to patients with wild-type *TERT* promoters (Figure 2E). Additionally, *hTERT* expression was significantly higher in tumors that used telomerase for maintenance of telomere length (*P* = .0002).

In our cohort, correlation with mutation analysis identified that *H3-3A* mutations are enriched in the group of tumors that use ALT as a mechanism of telomere maintenance (*P* = .0186). All the tumors harboring *H3-3A* G34R/V mutations were ALT positive and also had *TP53* mutations. Interestingly, several tumors demonstrated ALT activity in the absence of both *ATRX* and *H3-3A K*27M or G34R/V mutations.

## Discussion

Through an innovative randomized trial design, this phase II study compared the efficacy of 3 chemoradiotherapy regimens (vorinostat, bevacizumab, or temozolomide concurrent with RT), all with the same maintenance chemotherapy backbone of bevacizumab and temozolomide, for pediatric patients with newly diagnosed, localized cranial or spinal cord non-brainstem HGG, following an initial vorinostat feasibility cohort. Chemoradiotherapy with bevacizumab or vorinostat did not improve outcomes compared to concurrent temozolomide, and there was no significant improvement in long-term EFS and OS outcomes using the bevacizumab and temozolomide maintenance backbone, compared to contemporary pediatric HGG trials. Our results offer insight into the potential therapeutic role and vulnerability of telomerase maintenance mechanisms within pediatric HGG. Additionally, safety and feasibility of vorinostat combined with focal CNS RT were demonstrated, building on the previously reported experience with vorinostat in pediatric neuro-oncology,^30–32^ and establishing the same RP2D in non-brainstem HGG as has been described in diffuse intrinsic pontine glioma.^32^

There was no difference among the 3 chemoradiotherapy arms (vorinostat, bevacizumab, or temozolomide), demonstrating that neither vorinostat nor bevacizumab administered concurrently with RT improved outcomes compared to temozolomide. One-year EFS for all patients enrolled in the phase II study, with a maintenance backbone of temozolomide and bevacizumab, was 47.7%, consistent with outcomes reported in previous and contemporary clinical trials for non-brainstem HGG (Table 2), specifically (1) COG ACNS0126 single-arm phase II trial of concurrent temozolomide with RT followed by adjuvant temozolomide (1-year EFS: 37%),^1^ (2) COG ACNS0423 single-arm phase II trial of concurrent temozolomide with RT, followed by adjuvant temozolomide and lomustine (1-year EFS: 49%),^5^ and (3) HERBY randomized phase II trial of concurrent temozolomide with RT and adjuvant temozolomide, with or without bevacizumab (1-year EFS: 38% and 48%, respectively).^29^ Interestingly, patients enrolled on ACNS0822 who received temozolomide as chemoradiotherapy had nominally superior 1-year outcomes (EFS: 59%, OS: 85%) compared to the ANCS0126, ANCS0423, and HERBY trials using the same backbone.^1,5,29^ One-year OS for all patients in the present phase II study (77%) was also comparable to ANCS0126 (69%), ANCS0423 (72%), and HERBY (68% and 75% [with and without bevacizumab]). Notably, all these trials were developed prior to the current molecular era and therefore did not stratify treatment based on the presence of prognostically significant genetic alterations, such as *IDH*, or *H3-3A* mutations, which have been incorporated in contemporary study designs.

With over 5 years of follow-up (median follow-up: 59.9 months, range: 0.03–77 months), ACNS0822 provides valuable information on long-term survival outcomes. For all patients in the phase II study, 3-year EFS and OS are 14.8% and 24.9%, respectively, similar to ACNS0126 (11% and 20%)^1^ and ACNS0423 (22% and 28%).^5^ Importantly, 5-year EFS and OS are available from the ACNS0822 cohort, at 13.4% and 18.4%, respectively, and again slightly higher than ACNS0126 (8% and 12%) and slightly lower than ACNS0423 (14% and 22%). The are no significant differences in EFS and OS between ACNS0822 and the 2 predecessor trials.

Treatment on ACNS0822 was generally well tolerated. Vorinostat in combination with RT was associated with an increase in nausea and vomiting. Patients who were treated with temozolomide had more profound myelosuppression during chemoradiotherapy and maintenance therapy, as expected due to the cumulative bone marrow toxicity of alkylating agents, similarly described in ACNS0126 and ACNS0423.^1,5^ The reported toxicities with bevacizumab in ACNS0822 were limited and consistent with other published data.^29,33^ Among patients treated with bevacizumab, no grade 3/4 thromboembolic events were observed and a single patient had a Grade 3 intracranial hemorrhage. In contrast to the HERBY trial, which observed a significantly higher rate of adverse events, including proteinuria and arterial thromboembolism, in the bevacizumab arm leading to early discontinuation,^29^ use of bevacizumab was not associated with treatment cessation or excessive toxicities in ACNS0822.

Given the existing literature that suggests patients with HGG treated with bevacizumab may have an increased incidence of distant and diffuse disease at progression,^29,34^ we carefully analyzed patterns of progression in this study. Like the HERBY trial, the most common pattern of progressive disease for all patients was local progression. Although a higher proportion of patients receiving bevacizumab on the HERBY trial experienced both local and distant progression,^29^ we did not identify an increased risk of distant relapse in the subset of patients who received bevacizumab as chemoradiotherapy on ANCS0822.

In our study, histologic diagnosis impacted prognosis, with more favorable outcomes among patients with anaplastic astrocytoma when compared to patients with glioblastoma, as had previously been shown in ACNS0423.^5^ This is in contrast to the lack of prognostic value of histologic grade observed in the HERBY trial,^35^ though both demonstrated distinct molecular subgroups of HGG with variable outcomes, which are likely explained predominantly by genomic features.^36^

As this trial was designed and executed prior to recent discoveries elucidating the molecular landscape of pediatric HGG, comprehensive molecular profiling (such as whole exome or genome sequencing, or methylation array) were not incorporated; however, results from targeted sequencing and immunohistochemistry still provide valuable insight. Our study is the first to demonstrate inferior survival in tumors characterized by LOH at 10q and 17p. This association has been previously shown in adult glioma,^37^ and the potential adverse prognostic impact of 10q LOH was suggested by data from the Children’s Cancer Group 945 study.^38^ Taken together with worse outcomes observed in tumors harboring *PTEN* (located on chromosome 10q) and/or *PIK3CA* mutations seen in the present study, and recent data demonstrating prevalence of these alterations in some pediatric HGG,^9,36^ activation of PI3K/AKT/mTOR signaling pathway is likely driving a subset of HGG and may serve as a therapeutic target.

We also evaluated the prognostic impact of MGMT status in pediatric HGG. The lack of association between MGMT protein expression by immunohistochemistry and outcomes in this study contrasts with ACNS0126.^1^ Similarly, no correlation between *MGMT* promoter methylation and survival was observed in the ACNS0822 cohort, corroborating findings from the HERBY trial.^29,36^

This study also explored telomere maintenance mechanisms in pediatric HGG within the context of a clinical trial. Telomere dysregulation has been implicated in gliomas, which maintain telomere length to avoid senescence and apoptosis, which is induced by the replicative shortening of their chromosomes during cell division.^16–18^ While this is commonly achieved by re-activating telomerase, telomeres can also be maintained by ALT pathway which involves DNA repair and recombination. The ALT phenotype is more prevalent in pediatric glioblastoma compared to adult glioblastoma.^16–18^ These findings are supported by the present study in which 40% of evaluable HGG specimens displayed ALT activity only as a mechanism for telomere maintenance. Interestingly, our study identified 4 patients with an unidentified telomere maintenance mechanism (ALT and telomerase negative) with 100% EFS. Conversely, 2 patients with ALT- and telomerase-positive tumors had a significantly shorter OS compared to the rest of the cohort. Further research is necessary to better understand this finding and its potential impact on response to therapies, especially considering ALT is implicated in RT resistance in human glioma stem cells.^39^

Our study identified enrichment of ALT phenotype in HGGs with *H3-3A* mutations, consistent with prior reports.^15,40^ In the ACNS0822 cohort, all *H3-3A*-mutant tumors used ALT as a telomere maintenance mechanism, and most had co-occurring *TP53* mutations. Interestingly, 2 patients had *hTERT* promoter mutations, which are more commonly seen in adult gliomas, with inferior survival outcomes. One patient’s tumor with ALT phenotype as the sole telomere maintenance mechanism had no *H3-3A* mutations but harbored both *ATRX* and *IDH* (R132C) mutations, suggesting that *IDH* alterations, in cooperation with ATRX loss, may alternatively drive the ALT pathway, as previously described.^41^

Although previous studies have demonstrated potential connections between *ATRX*/*DAXX* mutations, *H3-3A* G34R/G34V mutations and ALT use, 6 patients with HGG in our cohort with ALT phenotype did not harbor H3.3K27M or G34R/V and/or *ATRX* mutations, suggesting acquisition of these alterations are not necessary or sufficient for the induction of ALT in HGG. Similar presence of ALT in pediatric HGG in the absence of ATRX mutations has recently been reported,^42^ though continued investigation in a larger cohort is needed to further elucidate the mechanistic biology.

In conclusion, chemoradiotherapy with vorinostat or bevacizumab did not improve outcomes compared to concurrent temozolomide for children with newly diagnosed HGG all treated with maintenance bevacizumab and temozolomide, with similar survival results as prior and contemporary clinical trials, highlighting the need to develop novel, biologically rational strategies. Results confirm the prognostic relevance of molecular alterations within distinct subgroups of pediatric HGG, for which future clinical trials should be stratified and designed. The role of telomere maintenance mechanisms in pediatric HGG tumorigenesis, co-occurring genetic alterations, and impact on survival were also demonstrated, supporting ongoing evaluation of telomerase- and telomere-based therapeutic interventions for HGG.

## Supplementary Material

vdae035_suppl_Supplementary_Material

## Data Availability

Deidentified data on which this research relies can be made available to the Journal for inspection and verification during the peer-review process.
