# Supplementary material for: Vorinostat, temozolomide or bevacizumab with irradiation and maintenance BEV/TMZ in pediatric high-grade glioma: A Children’s Oncology Group Study
Source: Neurooncol Adv. 2024 Mar 14;6(1):vdae035. doi: 10.1093/noajnl/vdae035 (PMC11003537; doi:10.1093/noajnl/vdae035)
Supplement: vdae035_suppl_Supplementary_Material [file vdae035_suppl_supplementary_material.docx]

**ACNS0822 Statistical Design**

This is a study for patients with newly diagnosed high-grade gliomas. Patients will be treated with local irradiation with a particular radiosensitizer, followed by maintenance chemotherapy with bevacizumab and temozolomide. The study has a feasibility part to obtain the MTD of vorinostat when given concurrently with RT. Upon its successful completion, the study will open to the Phase II part where patients will be randomized to receive concurrent radiation with 1 of the 3 radiosensitizers: vorinostat, bevacizumab, or temozolomide, and maintenance chemotherapy with bevacizumab and temozolomide. In the Phase II part, temozolomide is considered the control treatment; vorinostat and bevacizumab are considered experimental treatments. If at least one of the experimental treatments has a higher 1-year EFS than temozolomide in the Phase II part, the Phase III part of the study will open and the experimental treatment with a higher 1-year EFS between the 2 experimental treatments will be chosen for Phase III. Phase III of the study will formally compare the experimental treatment identified in Phase II to treatment with temozolomide. For the Phase II/III parts, randomization will be stratified by extent of resection (near total resection or gross total resection vs. other) and histology (glioblastoma multiforme vs. other). More details on each part of the study are discussed below.

The feasibility part will enroll 6 patients at 230 mg/m^2^/dose with daily RT. If there are fewer than 2 patients with DLTs, we will declare this dose of vorinostat as the recommended Phase II dose to be used with RT. If we have 2 or more DLT in this cohort of 6 patients, we would then dose de-escalate to 180 mg/m^2^ and open the cohort to 3 patients at this dose level. If 0 or 1 of the 3 patients has a DLT, we will expand this cohort by adding 3 additional patients. If fewer than 2 of the 6 patients have a DLT, this dose level (180 mg/m^2^) will be declared as the recommended Phase II dose. If 2 or more of the 6 patients at 180 mg/m^2^ have a DLT, the study will be amended. The probability that a dose level will be considered tolerable (ie, 0 or 1 DLT among 6 patients) is 0.89, 0.66, 0.42, or 0.23 if the true rate of DLT at that dose level is 10%, 20%, 30%, or 40%, respectively.

Upon successful completion of the feasibility part, the study will open for the Phase II part, with 108 patients randomized to one of the three treatments (36 patients per arm): vorinostat, bevacizumab, or temozolomide. At the end of the Phase II part, if neither of the 2 experimental arms has a 1-year EFS rate that is higher than that of the temozolomide arm, the study will be closed. If 1 of the 2 experimental arms has a 1-year EFS rate that is higher than that of the temozolomide arm, the study will continue to the Phase III part and this experimental arm will be chosen for comparison with temozolomide in the Phase III part of the study. If both experimental arms have a higher 1-year EFS rate than that of the temozolomide arm, the experimental treatment with the higher 1-year EFS rate will be chosen for further comparison with temozolomide. If the study continues to the Phase III part, patients will be randomized between 2 arms: one control arm (temozolomide) and one experimental arm. In the Phase III part, accrual will continue until 64 additional patients are randomized to each of the 2 arms. The final Phase III comparison will include patients randomized to the 2 arms in both the Phase II part and Phase III part of the study.

**Patient Accrual and Expected Duration of Trial**

Recent COG studies of patients with newly diagnosed high-grade gliomas (HGG) include ACNS0126 and ACNS0423. ACNS0126 accrued 99 eligible HGG patients between December 2002 and October 2004, with an average accrual rate of 54 patients per year. ACNS0423 accrued 107 eligible HGG patients between March 2005 and August 2007; the average accrual rate after the first 6 months was also about 54 patients per year. Therefore, the estimated annual accrual for the current study is 54 patients.

The feasibility part of the study will accrue 6-12 eligible patients. Given the overall annual accrual rate estimate of 54 patients, accruing 6-12 patients should take less than 6 months. Consider the 8 weeks evaluation period after the first 6 patients, or also after the first 9 and 12 patients, the complete duration for the feasibility part is expected to be about 12 months. The Phase II part of the study will accrue 108 eligible patients which is expected to take about 2 years. One year of additional follow-up will be needed to evaluate the Phase II outcome. If the study opens to the Phase III part, we will accrue an additional 128 eligible patients, which is expected to take about 2.4 years. Therefore, the total accrual duration (only the time when the study is open to accrual), if the study continues to the Phase III part, will be about 5 years. The total study duration from study activation until the end of accrual (including evaluation periods for the feasibility/II parts), is expected to be about 6.5 years. A minimal 1-year of additional follow-up is planned before the final Phase III analysis can be performed.

Of the 118 patients enrolled on the previous HGG study ACNS0423, 12 (10%) were deemed ineligible.  As of Amendment #1, the accrual for the feasibility part of the study was 6 patients and all were eligible. Considering a 10% rate of ineligible enrollment on this study, the total accrual for Phase II may be up to 120 patients and the total accrual for Phase III may be up to 142 patients.

**Statistical Analysis Methods**

Endpoints

Safety and feasibility endpoints:

- Feasibility part: DLT for vorinostat, when given concurrently with RT.
- Phase II: toxic death, individual toxicities especially Grade 3 or 4 hemorrhage, significant delay (> 2 weeks) in the completion of RT, and significant delay (> 2 weeks) in the start of any course of maintenance therapy.
- Phase III: toxic death, individual toxicities especially Grade 3 or 4 hemorrhage

Efficacy endpoints for Phase II/III:

- Event-free survival (EFS), defined as time to the first occurrence of any of the following events: disease progression, relapse, second malignant neoplasm, or death from any cause.
- Overall survival (OS), defined as time to death from any cause.
- Progression-free survival (PFS), defined as time to the occurrence of disease progression or recurrence.

Power Considerations for Phase II/III

In the Phase II part of the study, patients are randomized to 1 of the 3 arms: 1 control arm (temozolomide) and 2 experimental arms (vorinostat, bevacizumab). At the end of the Phase II part, the experimental treatment with a higher 1-year EFS rate among the 2 experimental arms, if its 1-year EFS is also higher than that of the temozolomide arm, will be chosen for further comparison with temozolomide in the Phase III part. If temozolomide arm has the highest 1-year EFS among the 3 arms, the study will be closed. We use 1-year EFS as the main endpoint for the Phase II part because, in this study, tumor response and shorter-term EFS are not considered good indicators of treatment effect.

ACNS0126 and ACNS0423 both used temozolomide and concurrent radiation therapy to treat patients with newly diagnosed high-grade gliomas. The maintenance therapies used in these 2 studies and in the current study are all different. Based on November 2008 data, the observed 1-year EFS (±SE) on ACNS0126 is 39% ± 5%; the observed 1-year EFS (±SE) on ACNS0423 is 49% ± 5%. Given these data, for power considerations we assume a 1-year EFS rate of 45% for the temozolomide arm (control arm) on this study. For the Phase III part of the study which will compare the overall EFS curves for the 2 arms, we assume a long-term EFS rate of 15% for the temozolomide arm, based on limited long-term data on ACNS0126 and long-term outcome data on an earlier high-grade glioma protocol CCG 945.

Between the 2 experimental arms, we use arm 1 to denote the arm with the better EFS in truth and arm 2 to denote the other. Below we discuss the probability of selecting arm 1 after the Phase II part and the probability of establishing its superiority in EFS over temozolomide at the end of the Phase III part. Obviously such probabilities depend on the true EFS of arm 1 and that of temozolomide arm. However, since the design only picks the better performer of the 2 experimental arms from the Phase II part, 1-year EFS for arm 2 will also influence the probability of selecting arm 1 after the Phase II part, and in term affect the probability of establishing the superiority of arm 1 over temozolomide at the end of the Phase III part.

In the table below, the selection probabilities for arm 1 and arm 2 after the Phase II part are presented in columns 2 and 3. For example, if the 1-year EFS rates for arm 1 and arm 2 are 60% and 40% respectively, the probability that the study will continue to the Phase III part with arm 1 is 0.85. On the other hand, if the 1-year EFS rate for arm 1 is 60% but the 1-year EFS rate for arm 2 is 55%, the probability of selecting arm 1 is reduced to 0.63, because under this scenario the chance of selecting arm 2 increases even though arm 1 is still better than arm 2 in truth. The probability of the study not continuing on to the Phase III part, meaning that neither of the 2 experimental arms has a 1-year EFS rate that is higher than that of the temozolomide arm, is one minus the probabilities in column 2 and column 3. In these scenarios, the 1-year EFS of arm 1 needs to be at least 60% compared to 45% in temozolomide arm for arm 1 to have a decent probability of being selected for the Phase III part. The probability of selecting arm 1 decreases with increasing 1-year EFS in arm 2.

**Supplementary Table 1: Probability of selection (after Phase II) and that of establishing superiority to temozolomide (after Phase III) for each experimental arm**

| **Scenarios** | **Phase II** Selection Probability** | | **Overall Phase III*** Success Probability** | |
| --- | --- | --- | --- | --- |
|  | arm 1 | arm 2 | arm 1 | arm 2 |
| arm 1 = 65%* |  |  |  |  |
| arm 2 = 60% | 0.66 | 0.32 | 0.65 | 0.30 |
| arm 2 = 55% | 0.78 | 0.18 | 0.77 | 0.13 |
| arm 2 = 50% | 0.88 | 0.09 | 0.86 | 0.03 |
| arm 2 = 45% | 0.91 | 0.04 | 0.90 | 0.01 |
| arm 2 = 40% | 0.94 | 0.01 | 0.92 | <0.01 |
|  |  |  |  |  |
| arm 1 = 60%* |  |  |  |  |
| arm 2 = 55% | 0.63 | 0.31 | 0.57 | 0.21 |
| arm 2 = 50% | 0.74 | 0.17 | 0.66 | 0.07 |
| arm 2 = 45% | 0.81 | 0.08 | 0.73 | 0.01 |
| arm 2 = 40% | 0.85 | 0.03 | 0.76 | <0.01 |
| arm 1 = 45%* |  |  |  |  |
| arm 2 = 45% | 0.31 | 0.31 | 0.03 | 0.03 |
| arm 2 = 40% | 0.38 | 0.17 | 0.04 | <0.01 |

*Temozolomide arm is the control arm, with 45% 1-year EFS.

**36 patients in each of the 3 arms.

***100 patients in each of the 2 arms including the 36 patients from the Phase II part.

Note that an experimental arm is only chosen for further study in the Phase III part if its 1-year EFS rate is higher than that of the temozolomide arm; if its outcome equals that of the temozolomide arm, it will not be chosen. In these calculations, for the rare situation where the 1-year EFS is the same for the 2 experimental arms and better than that of the temozolomide arm, half of that probability is added to the selection probability of arm 1 and half to that of arm 2. This simple approach means that, in the event of such a “tie”, we will randomly pick one experimental arm for further study. However, we will most likely consider other factors, such as toxicity profiles, for tie-breaking if such a tie occurs. The probabilities of such a tie are so rare that they have little impact on the results presented above.

For the Phase III part, a log rank test will be performed to compare the EFS between the 2 arms (100 patients each) after a minimal of 1 year of additional follow-up at a 1-sided alpha level of 0.05. However, for this study, the traditional power and size for this test between the chosen experimental arm and the temozolomide arm cannot be easily defined. This is because the occurrence of the Phase III part depends on the outcomes in the Phase II part which also involves the non-chosen experimental arm. In addition, the Phase II patients are included in the Phase III comparison; their early outcomes (1-year EFS) are already observed and have to occur in a particular direction (experimental arm higher than temozolomide) for the Phase III part to happen.

In column 4 and 5 of Table 1, we illustrate the overall probability of establishing a significantly better outcome for arm 1 (or arm 2) at the end of the Phase III with the specified log rank test in the current study design. For example, the success rate for arm 1 (with 60% 1-year EFS) ranges from 0.57- 0.76 if arm 2 has a 1-year EFS that is between 55% and 40%. The probability presented is the overall probability for a particular arm before the inception of the Phase II/III part, so is not conditional on the arm being chosen for Phase III. Note that if this is a standard Phase III comparison with the same sample size and other parameters, the power for the comparison between arm 1 and the temozolomide arm will be about 0.84. Here, the reduction in “power” is because arm 1, though the best of the 3 in truth, is not always chosen after the Phase II part, which reduces its overall success probability. Comparing column 2 and column 4, one can see that if arm 1 is truly the best of the 3 and “wins” in the Phase II part, the conditional probability of its overall success given the Phase II success and Phase II outcome will be quite high. In Table 1, selection probabilities for the Phase II part are computed by exact binomial probabilities. The overall success rates for the final Phase III comparison are estimated from simulation studies because the log rank test compares the entire EFS curve rather than just 1-year EFS. To carry out the simulations, additional assumptions need to be made on the failure patterns of the patients. Event-free survival curves are approximated by cure models where a proportion of the patients become long-term survivors and failures for the remaining patients follow an exponential pattern. The temozolomide arm is assumed to have 45% 1-year EFS and 15% long-term EFS. The whole EFS curve under the cure model with exponential failure is fully specified by EFS rates at these 2 time points. The differences in event free survival between the temozolomide arm and the experimental arm are assumed to be the same at 1 year and at long term, which then specify the whole EFS curves for the experimental arms.

**Interim Monitoring**

Monitoring for Toxic Death and Grade 3 or 4 Hemorrhage

During the Phase II part of the study, we will monitor the rate of toxic death and that of Grade 3 or 4 hemorrhage separately for each arm during RT. In ACNS0126, among 99 patients there were 2 deaths within 1 month on study, which are considered slightly attributable to the treatment. In ACNS0423, all 7 deaths which occurred during treatment or within 31 days after treatment are considered due to disease progression. Two incidences of Grade 3 or 4 hemorrhage were reported among 105 patients during chemoradiotherapy on ACNS0423. Based on these data, for the current study we consider 2% the maximum tolerable rate for both toxic death and for Grade 3 or 4 hemorrhage. For each arm, we will accept at most 2 toxic deaths or 2 incidences of Grade 3 or 4 hemorrhage among the 36 patients. The monitoring rule will be triggered 3.5%, 37%, 56%, or 82% of the time if the true incidence is 2%, 6%, 8%, or 12%, respectively. We will also monitor toxic death rate and Grade 3 or higher hemorrhage rate separately during maintenance therapy for patients from all 3 arms combined, since they all receive the same maintenance therapy. The rule for maintenance therapy will be triggered if at least 4/54, 6/108 patients experience toxic death/hemorrhage; it is satisfied 4% of the time if the true incidence is 2%, and 80% of the time when the true incidence is 7%.

If the study opens to the Phase III part, the rate for toxic death and that for Grade 3 or 4 hemorrhage during chemoradiotherapy will be also examined for each arm. Among the 100 patients on each arm (temozolomide arm and the chosen experimental arm), we can accept at most 4 incidences for toxic deaths or for Grade 3 or 4 hemorrhage. The chance that the combined rule for Phase II/III (≥3/36, ≥5/100) will be satisfied is 7% if the true incidence is 2%, and is 85% if the true incidence is 7%. The rate of toxic death and that for Grade 3 or 4 hemorrhage during the common maintenance will also be examined by including all patients treated on Phase II or Phase III parts. We will consider the rate for toxic death or that for Grade 3 or 4 hemorrhage unacceptable if we observe 9 or more incidences among the 236 Phase II/III patients. The combined rule (≥4/54, ≥ 6/108, ≥ 9/236) will be satisfied 7% of the time if the true incidence is 2%; it will be met 85% of the time when the true incidence is 5%.

When a safety monitoring rule is met, the study accrual will be temporarily suspended. The study committee will perform a careful review of the toxicity incidences, and report the results and study committee recommendation such as accrual suspension or therapy modification for the involved arm to the DSMC. DSMC also may make other recommendations based on their review of the toxicity and safety data at any time.

Monitoring for Feasibility of Each Radiosensitizer and Common Maintenance Chemotherapy

During the Phase II part of the study, the feasibility of each radiosensitizer and the common maintenance therapy will be examined via the incidence of significant delay (> 2 weeks) in the completion of RT and the incidence of significant delay (> 2 weeks) in the start of any course of maintenance (among total courses combining all 3 arms). A review of treatment feasibility and patient safety will be undertaken when we observe > 10% significant delays in the completion of RT specifically if we observe at least 5/18 or 8/36 significant delays. The rule will be met about 4% of the time if the true incidence of delay is 10%, and 90% of the time if the true incidence is 30%. Because the number of total maintenance courses cannot be easily estimated (as patients may not all complete 12 courses), it is not feasible to specify the set of statistics-based boundaries for monitoring delays in maintenance therapy. The monitoring will be based on the marginal incidence of significant delay in the start of any course of maintenance observed at each interim report. Similar review will be undertaken if the rate of delays during maintenance exceeds 10%.

Efficacy monitoring

At the end of the Phase II part, interim comparisons of EFS by log rank test will be performed to evaluate whether there is early compelling evidence of improvement in EFS in either experimental arm compared to the control arm. The monitoring boundary will be based on Lan-Demet’s method with spending function αt^2^. If there is significant evidence of efficacy for either experimental arm compared to the control arm, the study will be referred to DSMC for consideration of early closure after the Phase II part due to efficacy. If the study continues to the Phase III part, an interim comparison of EFS by log rank test between the chosen experimental arm and the control arm will be performed approximately halfway through the Phase III enrollments and will be based on Lan-Demet’s method with spending function αt^2^. In addition, during Phase II/III, for each interim Data and Safety Monitoring Committee (DSMC) report, outcome of the current study participants will be compared to historical data on ACNS0126 and ACNS0423. This informal monitoring aims to compare outcome of patients who received maintenance therapy with bevacizumab to patients who did not receive maintenance therapy with bevacizumab. Since such comparison is not an aim of the study, we will not employ any formal monitoring rules; if, in an interim analysis, the outcome of the current study is significantly worse than those of the prior studies (with a p value of < 0.05), the issue will be brought to the DSMC for their attention and evaluation.

**Analysis Plan**

Analysis plans for the primary aims of Phase II/III

We will estimate event-free survival (EFS) for patients on each arm by Kaplan-Meier product-limit method. After the Phase II part, the experimental arm with a higher nominal 1-year EFS will be chosen for further study in the phase III part if its 1-year EFS rate is also higher than that of the temozolomide arm. If the study opens to the Phase III part, Kaplan-Meier curves will be used to estimate EFS of the 2 arms combining all patients randomized to the regimen during the Phase II and the Phase III parts. The primary analysis will be based on a log rank test that compares the EFS between the chosen experimental arm and the temozolomide arm; the cutoff value for the Z-statistics will be 1.64.

Analysis plans for Phase II/III secondary aim

Event-free survival (EFS), overall survival (OS), and progression-free survival (PFS) will be calculated for patients treated with each radiosensitizer by Kaplan-Meier estimates. Log rank tests will be used to compare these survival outcomes between different treatment groups on the current study. Similar log rank analyses will be performed to compare outcomes for patients treated on the current study to those for HGG patients treated on previous COG protocols (ACNS0126 and ACNS0423); in particular, outcomes for patients on Arm B (temozolomide) will be compared to that for HGG patients on ACNS0126 and ACNS0423 to isolate contribution of bevacizumab plus temozolomide maintenance therapy versus single agent temozolomide maintenance therapy and temozolomide plus lomustine maintenance therapy, respectively. Incidence of individual toxicities will be estimated for each chemoradiotherapy arm and for maintenance chemotherapy.

Analysis plans for laboratory and imaging aims

Descriptive statistics will be used to summarize the biological/laboratory/imaging measures for the study, including telomerase activity, hTert expression, telomere length, MGMT promoter methylation status, MGMT protein expression, and various measures in MR perfusion and diffusion from MR imaging. Changes in these measures across time-points (baseline, prior to maintenance therapy, prior to cycle 3 and after maintenance therapy) will be summarized similarly, using descriptive statistics on the measures that are collected at multiple time points. Mean and standard deviation will be used to summarize continuous measures; log transformation of a continuous measure may be considered when appropriate. Categorical measures will be described by percentage distribution of the categories; in the case of binary measures a 95% CI will be estimated. SNP analysis and gene expression and microRNA analysis will be carried out using Affymetrix software, and dChip or GCOS software respectively. Exploratory analyses to correlate a particular biological/laboratory/imaging characteristic with survival outcomes might be performed. Log rank tests will be used to explore the prognostic significance of a categorical factor on EFS, OS or PFS. Cox proportional hazards models will be used to explore the effect of a continuous marker on survival outcomes, and will be used for exploratory multivariate analysis examining the effect of the characteristic of interest with adjustments for other patient or treatment characteristics. The power of such exploratory analyses examining the association of a laboratory or imaging characteristics with survival outcomes depend on many factors: 1) total sample size of the study (whether the study goes to Phase III); 2) the portion of patients that participate in the particular optional study; 3) the proportion of patients possessing the high risk characteristics (assuming a simple comparison based on a binary risk factor); 4) the survival outcomes in the high-risk group and in the low-risk group. Therefore, it is difficult to prospectively estimate the power characteristics for individual biology analysis and these analyses are exploratory in nature.

**Gender and Ethnicity Considerations**

Review of outcome data from previous COG studies for high-grade glioma indicates that treatment effects are consistent within gender and ethnicity. That is, no one treatment examined has proven superior for one gender or ethnic group. Because of this, the study size will not be adjusted to ensure high power to detect differences in outcome in groups defined by ethnicity or gender.

**Gender and Minority Accrual Estimates**

The target for the feasibility part of the study was 6-12 eligible patients. As of Amendment #1, the accrual for the feasibility part of the study has been completed and was 6 patients and all were eligible. The expected accrual for the Phase II and Phase III parts of the study are 108 eligible patients and 128 eligible patients, respectively. The accrual target on the study, therefore, is 242 eligible patients. Considering a 10% ineligibility rate for Phase II and Phase III, the maximum enrollment for this study may be up to 268 patients in order to assure that the required number of eligible patients are enrolled.

The gender and minority distribution of the study population is expected to be:

| **Accrual Targets** | | | |
| --- | --- | --- | --- |
| **Ethnic Category** | **Sex/Gender** | | |
|  | **Females** | **Males** | **Total** |
| Hispanic or Latino | 11 | 9 | 20 |
| Not Hispanic or Latino | 111 | 137 | 248 |
| **Ethnic Category: Total of all subjects** | 122 | 146 | 268 |
| **Racial Category** |  | | |
| American Indian or Alaskan Native | 0 | 0 | 0 |
| Asian | 6 | 5 | 11 |
| Black or African American | 16 | 9 | 25 |
| Native Hawaiian or other Pacific Islander | 2 | 0 | 2 |
| White | 98 | 132 | 230 |
| **Racial Category: Total of all subjects** | 122 | 146 | 268 |

This distribution was derived from the recent COG study ACNS0423 for patients with newly diagnosed high-grade gliomas.
